# Hemodynamic Changes in the Carotid Artery after Infusion of Normal Saline Using Computational Fluid Dynamics

**DOI:** 10.3390/diagnostics10070473

**Published:** 2020-07-12

**Authors:** Ui Yun Lee, Chul In Kim, Gyung Ho Chung, Jinmu Jung, Hyo Sung Kwak

**Affiliations:** 1 Division of Mechanical Design Engineering, Jeonbuk National University, Jeonju 54896, Korea; euiyun93@naver.com (U.Y.L.); kci2850@jbnu.ac.kr (C.I.K.); 2 Department of Radiology and Research Institute of Clinical Medicine of Jeonbuk National University, Biomedical Research Institute of Jeonbuk National University Hospital, Jeonju 54907, Korea; chunggh@jbnu.ac.kr; 3 Hemorheology Research Institute, Jeonbuk National University, Jeonju 54896, Korea

**Keywords:** computational fluid dynamics, carotid artery, normal saline, blood viscosity, wall shear stress

## Abstract

Purpose: To study the effect of the infusion of normal saline on hemodynamic changes in healthy volunteers using computational fluid dynamics (CFD) simulation. Methods: Eight healthy subjects participated and 16 carotid arteries were used for the CFD analysis. A one-liter intravenous infusion of normal saline was applied to the participants to observe the hemodynamic variations. Blood viscosity was measured before and after the injection of normal saline to apply the blood properties on the CFD modeling. Blood viscosity, shear rate, and wall shear stress were visually and quantitatively shown for the comparison between before and after the infusion of normal saline. Statistical analyses were performed to confirm the difference between the before and after groups. Results: After the infusion of normal saline, decreased blood viscosity was observed in the whole carotid artery. At the internal carotid artery, the recirculation zone with low intensity was found after the injection of normal saline. Increased shear rate and reduced wall shear stress was observed at the carotid bifurcation and internal carotid artery. The hemodynamic differences between before and after groups were statistically significant. Conclusions: The infusion of normal saline affected not only the overall changes of blood flow in the carotid artery but also the decrease of blood viscosity.

## 1. Introduction

The flow resistance increases as the whole blood viscosity increases. There are several parameters that increase blood viscosity such as hematocrit, plasma viscosity, and temperature. In contrast, the infusion of normal saline makes hematocrit dilution. Due to the infusion of normal saline, whole blood viscosity and flow resistance become low [1,2].

Normal saline (0.9% NaCl solution) is generally used for intravenous infusion therapy in medical practice [3,4]. It has been reported that injection of normal saline into healthy subjects not only induces physiological changes (i.e., reduction of pH) but also causes functional variation of blood flow (i.e., hemodynamic factors) [5]. For example, normal saline infusion at the renal artery was reported to reduce the velocity of blood flow [6]. In contrast, increased velocity was found at the coronary artery after the injection of normal saline [7]. However, despite the fact that normal saline has been extensively used in clinical practice, there is still a lack of research on how the normal saline affects hemodynamic changes, such as wall shear stress and blood viscosity in blood vessels.

Due to the dynamic flow characteristics in the carotid artery bifurcation, a high wall shear stress usually appears at the carotid bifurcation, and a recirculation flow pattern with low wall shear stress can be found at the carotid bulb, which is prone to plaque formation [8,9,10,11,12]. According to the variation of blood viscosity and blood flow, it might be possible to change the wall shear stress at the carotid artery. Moreover, this hemodynamic variation plays an important role in clinical practice [13].

The recent studies have introduced several methods to analyze hemodynamic variations. For example, 4d flow is used to determine blood flow with velocity in cerebral artery [14,15,16], and the obtained wall shear stress and T1 contrast ratio using MRA are utilized to investigate the cause of carotid exposure in endarterectomy [17]. In addition, CFD analysis is conducted to analyze the aneurysm growth and the risk of aneurysm rupture risk based on MRI or MRA data [18,19,20]. The advantage of conducting computational fluid dynamics (CFD) simulation is to quantitatively and visually show the hemodynamic characteristics with respect to arterial disease [21,22,23]. For example, the large variation of wall shear stress at the carotid artery can be considered as the site of growth and progression of atherosclerosis [24,25,26,27]. Various studies for CFD simulation have been applied to predict blood flow in the carotid artery. For example, Cibis et al. [28] and Gharahi et al. [29] used non-Newtonian models with assumed value without actual measurement and observed wall shear stress and recirculation zone at the carotid bulb. The assumed blood viscosity has a limitation to provide actual patient-specific hemodynamic information in CFD analysis [30]. To overcome this limitation, this study utilized patient-specific geometry models with actually measured non-Newtonian blood viscosity values to apply CFD simulation for accurate prediction of blood flow in carotid artery.

The purpose of this study was to measure the change in non-Newtonian viscosity before and after the saline injection of volunteers and apply the measurement results as a parameter for CFD study to analyze changes in hemodynamic factors in blood vessels. We compared the hemodynamic factors (velocity, shear rate, blood viscosity, and wall shear stress) between before and after the infusion of the normal saline.

## 2. Materials and Methods

### 2.1. Subject Selection

This study was approved by the institutional review committee of Jeonbuk National University Hospital and was conducted with the informed consent from the volunteers (project identification code: 2017-10-007, approval date: 2017-10-07). From April 2017 to July 2017, we did magnetic resonance angiography (MRA) on eight healthy volunteers. The criteria for the healthy volunteers were no atherosclerosis in MRA and age below 40 years. Seven men and one woman participated, and the age range was 28–35 years (mean age: 30.25 years, standard deviation: 2.27 years). A total of 16 carotid arteries (left and right sides) were used for this study. Depending on the one-liter intravenous infusion of normal saline, the study group was classified into “before” and “after” infusion of the normal saline groups.

### 2.2. Image Processing

Imaging was performed with a 3T MRI system (Skyra; Siemens, Erlingen, Germany) with 16-channel wrist coils. We acquired images using only multi-slab three-dimensional time-of-flight MRA with the following parameters: TR = 23 ms; TE = 3.45 ms; flip angle = 20°; field of view = 200 mm; matrix size = 488 × 249; sensitivity encoding factor = 2.5; and slice thickness = 1.2 mm.

**Figure 1:**
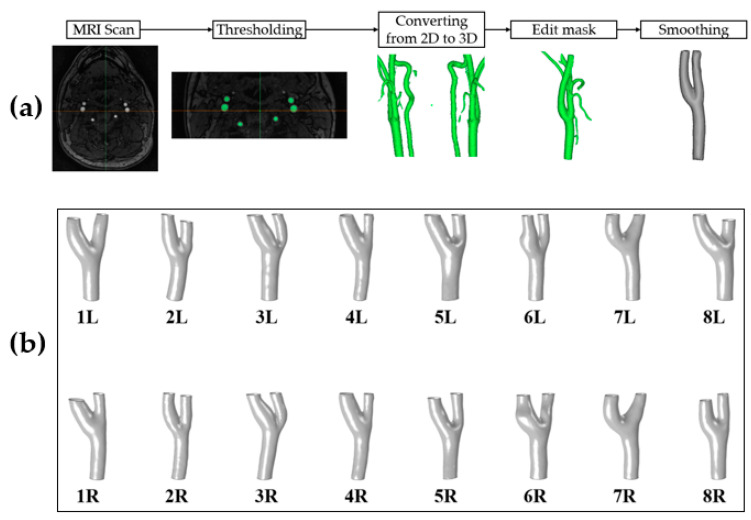
(**a**) The procedure of reconstruction for participant-specific carotid artery geometric models. See the text for the detailed description. (**b**) Participant-specific carotid artery geometric models of healthy volunteers (the top row, left internal carotid artery; the bottom row, right internal carotid artery).

The saved DICOM image files were converted from two-dimensional images into three-dimensional images using Mimics Software (version 20.0; Materialise NV, Leuven, Belgium). In the reconstructed geometry, unnecessary branches of the external carotid artery were truncated using an edit mask in a three-dimensional tool. The smoothing tool was performed for the final vessel model before the CFD simulation, as shown in Figure 1a. The top row shows the eight left carotid arteries, and the bottom row shows the eight right carotid arteries Figure 1b.

### 2.3. Blood Viscosity

We measured participant-specific non-Newtonian blood viscosity before injecting normal saline with a scanning capillary tube viscometer (Rheovis-01; Biorheologics Co., Ltd., Jeonju, South Korea). After injecting normal saline, we measured the blood viscosity for each volunteer again to compare the effect of normal saline on the viscosity change of the blood. Non-Newtonian blood viscosity was measured from a shear rate of 1 to 1000 s^−1^. For the CFD analysis, we calculated Casson constant and yield stress based on the obtained blood viscosity data.

### 2.4. Computational Fluid Dynamics 

The reconstructed three-dimensional carotid artery models were imported into COMSOL Multiphysics software (version 5.2a; COMSOL Inc., Burlington, MA, USA) for CFD simulation, and mesh was generated for analysis. Blood flow was calculated based on the Navier-Stokes equation (Equation (2)) and continuity equation (Equation (1)) [31]. In the Equations (1) and (2), *u* denotes flow velocity. In Navier-Stokes equation, ρ represents density, $\frac{\partial u}{\partial t}$ indicates the rate of change of velocity with time, *p* and μ stand for the pressure and fluid viscosity, respectively [32,33].

(1) ∇·u=0

(2) $$\rho \left(\frac{\partial u}{\partial t}+u\cdot \nabla u\right)=-\nabla p+\mu \nabla^{2}u$$

Laminar flow and incompressible condition were applied as boundary conditions. Using the actually measured whole blood viscosity, Casson constant and yield stress were obtained. The obtained parameters were applied to the non-Newtonian Casson model in the CFD analysis as follows:

(3) $$\sqrt{\tau }=\sqrt{\tau_{y}}+\sqrt{k}\sqrt{\dot{\gamma }}\;\mathrm{when}\;\tau >\tau_{y}$$

(4) $$\dot{\gamma }=0\;\mathrm{when}\;\tau <\tau_{y}$$

In the Equation (3), τ is wall shear stress, and $\tau_{y}$ denotes the yield stress. k indicates a Casson constant, and $\dot{\gamma }$ stands for the shear rate [34,35].

Due to the insufficient data on the vessel wall, a rigid with no-slip condition was applied to blood vessel [29]. For inlet flow condition, we used a flow waveform from the healthy subject using the ultrasonic measurement [36]. Traction-free was applied as the outlet flow condition [37]. We analyzed three cardiac cycles, and the last cardiac cycle was taken for the results [12].

### 2.5. Hemodynamic Analysis

Visualization results of blood viscosity, velocity, shear rate, and wall shear stress at end-diastole were obtained. We calculated the minimal, time-averaged, and maximal values of hemodynamic factors during the cardiac cycle. The left and right carotid arteries of hemodynamic variables were calculated, respectively. In addition, hemodynamic variables were obtained, not only the values for the whole carotid artery, but also those for specific regions of the internal carotid artery, which is known to be the site prone to endothelial cell dysfunction because of hemodynamic changes. The whole carotid artery was defined as the entire carotid artery except for one inlet and two outlets. Statistical analysis was performed for confirmation of the significant difference between before and after infusion of normal saline groups. Wilcoxon signed-rank test was used for the non-normally distribution variables. We employed a paired *t*-test for the normally distributed variables. We considered *p* < 0.05 as statistically significant.

## 3. Results

### 3.1. Blood Viscosity Change

**Figure 2:**
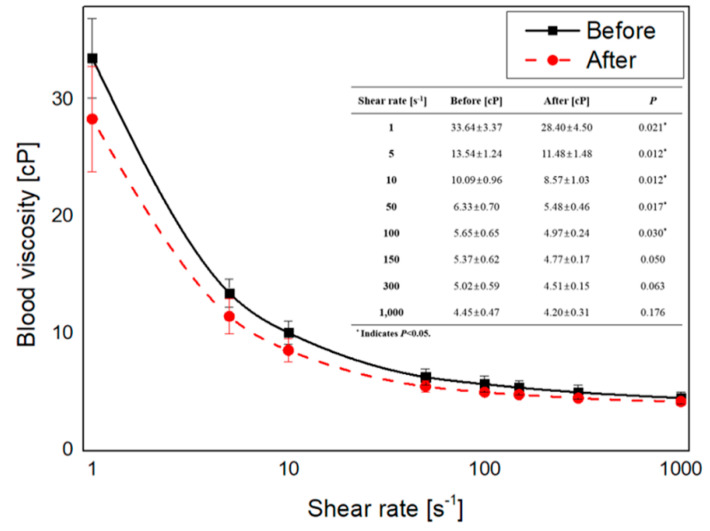
The measured whole blood viscosity characteristics of participants. The solid and dotted lines indicate participant-specific non-Newtonian blood viscosity profiles before and after normal saline infusion, respectively. The table inside the graph shows quantitative result of whole blood viscosity before and after infusion of normal saline at different shear rates. The whole blood viscosity values are expressed as the mean and standard deviation.

The participant-specific blood viscosity profiles were measured as shown in Figure 2. The results of blood viscosity were different depending on the shear rate. Overall, decreased blood viscosity was observed in the after group, whose average blood viscosity (28.40 ± 4.50 cP) at a shear rate of 1 s^−1^ was 18% lower than that of the before group (33.64 ± 3.37 cP) (*p* = 0.021). Approximately an 11% difference of blood viscosity at a shear rate of 300 s^−1^ was found, (5.02 ± 0.59 cP for the before group and 4.51 ± 0.15 cP for the after group). According to Jung et al.’s [34] reference interval of blood viscosity, in this study, the blood viscosity of two men and one woman before the infusion of blood viscosity was not included in the reference interval, but the results of blood viscosity after blood viscosity were involved in the reference interval.

**Figure 3:**
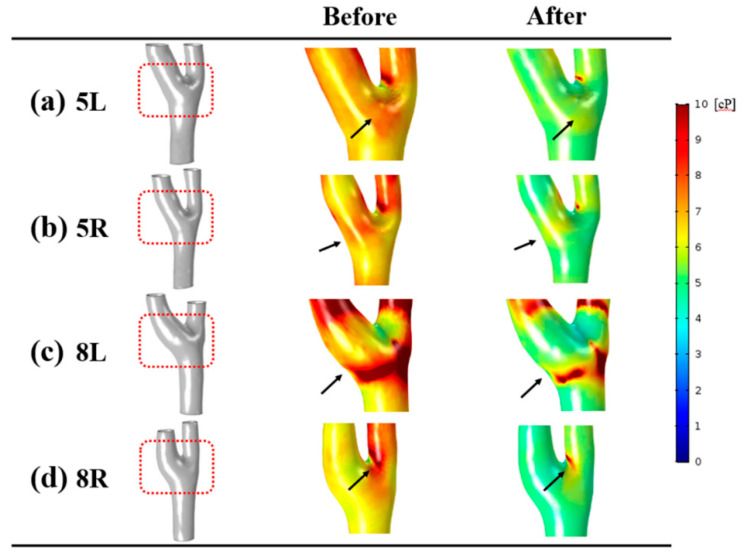
Distributions of blood viscosity at end diastole. Four representative cases were selected. The regions with large variations of blood viscosity are marked with black arrows.

Due to applying non-Newtonian blood viscosity, dynamic distributions of blood viscosity according to shear rate were observed. The blood viscosity between the before and after groups are compared in Figure 3. After infusion of normal saline, not only the blood viscosity of specific sites, such as the carotid bifurcation and internal carotid artery, but also the blood viscosity of the whole carotid artery became reduced at end-diastole. The dynamic variation of blood viscosity was found at the carotid bulb and is marked with black arrows. Before the injection of normal saline, highly increased blood viscosity appeared at the carotid bulb, and reduced blood viscosity was observed at the carotid bulb in after group.

### 3.2. Velocity

**Figure 4:**
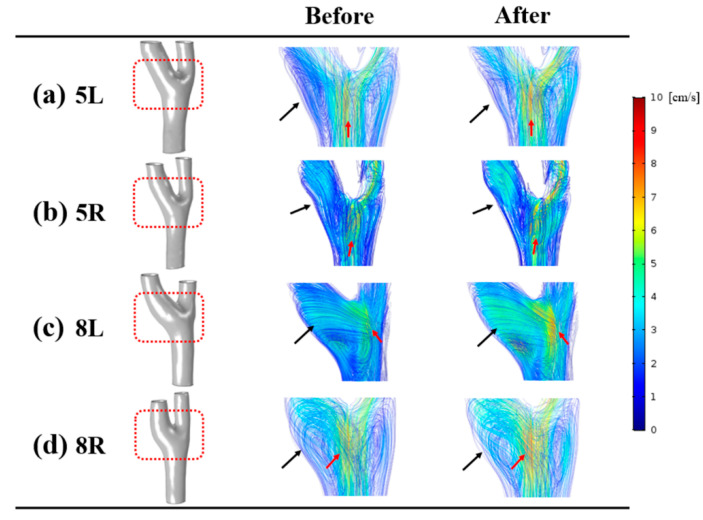
Distributions of velocity with streamlines. The enlarged region is highlighted with red boxes for each carotid artery model. A recirculation zone, marked with black arrows, was observed at the internal carotid artery due to the low velocity. Red arrows indicate the area with a change of velocity at the carotid bifurcation. See the text for a detailed description.

The distributions of velocity at end-diastole are shown in Figure 4. For comparison between the before and after groups, we selected four representative cases (left (5L and 8L) and right (5R and 8R)) for modelling. Overall, the blood flow at the recirculation area of the before group was highly intense. In contrast, blood flow with low intensity was observed at the recirculation zone of the after group. For example, whereas the 5L model of the before group had blood flow with high intensity at the internal carotid artery, the same model of the after group had blood flow with low intensity. In addition, after infusion of normal saline, the increased velocity was observed at the carotid bifurcation. The regions with large variations of velocity are pointed out with red arrows.

### 3.3. Shear Rate

**Figure 5:**
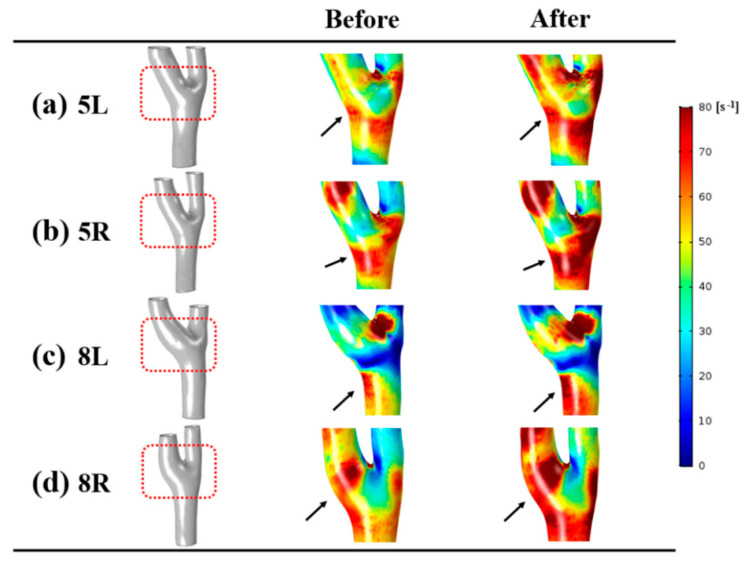
Distributions of shear rate at end-diastole. Before and after groups are shown with the representative cases. Changes of shear rate are indicated with black arrows. Increase of shear rate was found after the infusion of normal saline.

In Figure 5, the distributions of the shear rate at end-diastole are shown with the representative cases. Shear rate is defined as the rate of variation of deformation [38]. The same scale bar was applied for the accurate comparison between the before and after groups (0 to 80 s^−1^). After the infusion of normal saline, an increased shear rate was found where the recirculation zone with low intensity was observed compared to before group. All models of the after group had a greater shear rate at the carotid bifurcation than did the before group.

The minimal, time-averaged, and maximal shear rate of the before and after groups are quantitatively shown in Table 1. For the detailed comparison between the before and after groups, shear rate was calculated at the left and right sides of the carotid artery and internal carotid artery. The overall tendency showed that an increased shear rate was found after the infusion of normal saline regardless of the location of the carotid artery. In the whole carotid artery, minimal shear rate at the right side was significantly different (*p* = 0.036). At the internal carotid artery, the time-averaged shear rate of after group was statistically greater (*p* = 0.012 for left, *p* = 0.036 for right). The maximal shear rate at the left side of the internal carotid artery was also significantly higher than in the before group (*p* = 0.012).

### 3.4. Wall Shear Stress

**Figure 6:**
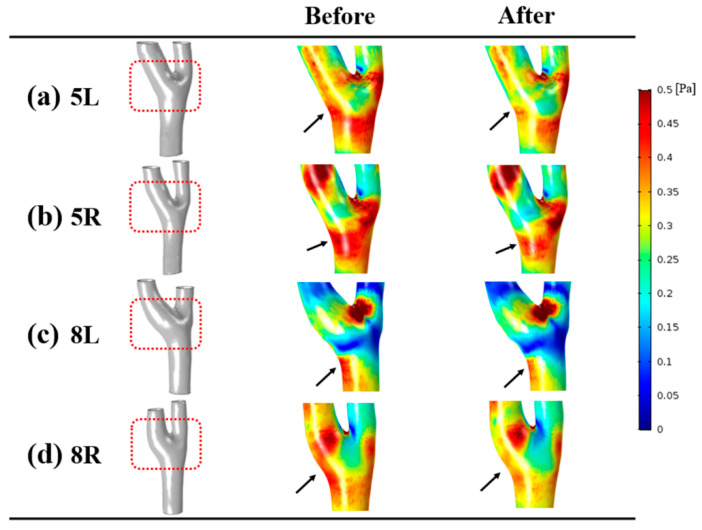
Distributions of wall shear stress at end-diastole. After infusion of normal saline, reduction of wall shear stress was observed at the carotid bulb, marked with black arrows.

**Figure 7:**
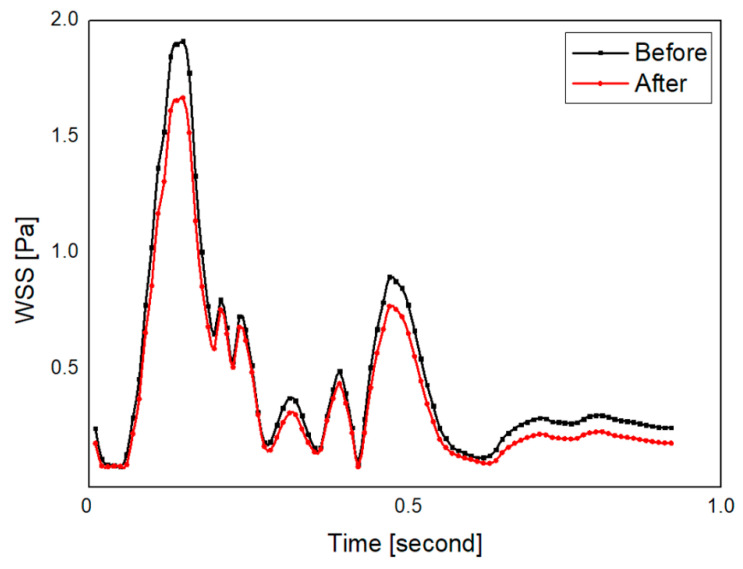
Wall shear stress (wss) profile of right internal carotid artery during cardiac cycle of the representative case (model 8). After the injection of normal saline, WSS becomes lower.

The distributions of wall shear stress at the end-diastole was compared between the before and after groups as shown in Figure 6. The scale bar for wall shear stress was set from 0 to 0.5 Pa. After the infusion of normal saline, the wall shear stress was found to be reduced where the recirculation was observed. The region with reduced wall shear stress is indicated with black arrows. Mostly, decreased wall shear stress appeared at the internal carotid artery, and no large variation of wall shear stress was found at the carotid bifurcation. Wall shear stress became reduced after injection of normal saline at both sides of the whole carotid artery and internal carotid artery, as shown in Table 2. A statistically significant difference was observed between the before and after groups at both sides of the whole carotid artery. For the wall shear stress at the internal carotid artery, the right side of minimal, time-averaged, and maximal wall shear stress and the left side of minimal wall shear stress showed a significant difference between the two groups. At the left side of the whole carotid artery, the time-averaged wall shear stress of the before group (0.92 ± 0.18 Pa) was 9% greater than that of the after group (0.84 ± 0.19 Pa). At the left side of the internal carotid artery, the minimal wall shear stress of the after group (0.08 ± 0.02 Pa) was 12% lower than that of the before group (0.09 ± 0.01 Pa). A graph of wall shear stress of right internal carotid artery during cardiac cycle is shown in Figure 7.

## 4. Discussion

In this study, we examined flow characteristics of the carotid artery after infusion of normal saline. We came up with four major findings. First, we found a significant reduction of blood viscosity at the whole carotid artery in the after group. Second, we observed a recirculation zone with high intensity at the internal carotid artery in the before group, and a recirculation region with low intensity appeared in the same area after injection of normal saline. Third, the after group had increased shear rate at the internal carotid artery and carotid bifurcation compared to before group. Lastly, reduced wall shear stress was observed at the internal carotid artery after the infusion of normal saline.

### 4.1. Normal Saline

According to Sherif et al.’s study [39], normal saline has been used for a long time and is widely used in clinical practice. Tim et al. [40] reported that, although most of the people recognized that intravenous fluid therapy was useful for health, a response to the basic question, such as when, what, and how (much) regarding the fluid therapy, was challenging. Therefore, studies are needed to suggest guidelines on fluid therapy. However, Neil et al. [4] argued that normal saline might cause side effects in a clinical study. Therefore, the use of Plasma-Lyte A might be better to use although it is more expensive compared to normal saline (0.9% NaCl solution). Since normal saline could trigger side effects, in vitro studies that validate the effect of injecting normal saline are required [4,39,40]. In this study, we investigated the blood flow characteristics in healthy carotid arteries after infusion of normal saline. The blood viscosity was relatively reduced after applying normal saline. The blood flow at the recirculation area with low intensity was found, and a decrease of wall shear stress was observed in the after group.

### 4.2. Velocity

Birchall et al. [11] conducted CFD studies on normal and diseased carotid arteries. The blood flow from the common carotid artery mainly flowed at the center of the vessel instead of at the wall. Due to the carotid bifurcation, blood flow was divided separately into the internal carotid artery and the external carotid artery. Therefore, the low velocity at the wall of the internal carotid artery could be observed. Compared to Birchall et al.’s research, we showed a similar streamline in the carotid artery. In this study, recirculation area with high intensity was observed at the carotid bulb before infusion of normal saline, and the recirculation zone with low intensity was found after infusion of normal saline.

### 4.3. Hemodynamic Characteristics at the Carotid Bulb

Li et al. [10] regenerated the stenotic carotid artery to construct a normal carotid artery. They studied changes of hemodynamic variables (velocity, wall shear stress, and recirculation zone) at the carotid bulb. Relatively low velocity, wall shear stress, and recirculation zone was found at the carotid bulb. These hemodynamic characteristics might be related to the formation and growth of atherosclerosis. In this study, a relatively low velocity, wall shear stress, and recirculation zone at the regions of a healthy carotid bulb were observed regardless of fluid injection compared to the remaining areas of carotid artery. Notably, after the infusion of normal saline, the relative tendency at the carotid bulb was decreased more. These results were consistent with many researchers’ studies, including that of Li et al. [9,41].

According to recent study, the high wall shear stress is associated with the risk of cerebrovascular disease [17]. In this study, we observed the effect of improving the overall wall shear stress of carotid artery after injection of normal saline. For example, the blood viscosity was 18.45% lower at shear rate 1 s^−1^ after the infusion of normal saline, and the time-averaged wall shear stress of right internal carotid artery was 8.33% lower after the injection of normal saline. The quantitative results of wall shear stress were similar to Cibis et al.’s study [28] and Long et al.’s study [42]. According to Cibis et al.’s study, the average wall shear stress was 0.73 Pa ± 0.25, and the average values of wall shear stress of our study were 0.50 and 0.52 Pa for the left and right internal carotid artery, respectively. Moreover, Long et al. found that the range of wall shear stress near the bifurcation was from 0 to 1.5 Pa, and the wall shear stress results of this study were also included in the same scope.

### 4.4. Blood Viscosity

Webb et al. [43] obtained blood samples from healthy people to conduct an in vitro experiment. Normal saline (0.9% NaCl solution) was added to the collected blood samples, and the blood viscosity was measured. As a result, decreased blood viscosity was observed. In this study, we injected normal saline (0.9% NaCl solution) directly into the vein of healthy volunteers and analyzed the changes of blood flow using CFD by applying the measured participant-specific non-Newtonian blood viscosity. Although the method was different from Webb et al.’s research, the change of blood viscosity according to applying normal saline showed the same results.

### 4.5. Relationship between the Hemodynamic Factors

Box et al. [13] studied the effect of non-Newtonian blood viscosity on wall shear stress by modeling a carotid bifurcation model. They controlled hematocrit and plasma to adjust the difference of blood viscosity and performed CFD analysis. They found that lowering the hematocrit resulted in low blood viscosity, high shear rate, and low wall shear stress. The present study has yielded hemodynamic results similar to those of Box et al.’s study. In this study, participant-specific carotid artery showed decreased viscosity, increased velocity, shear rate, and reduced wall shear stress after infusion of normal saline.

### 4.6. Limitations

There were two limitations in this study. First, only eight volunteers were involved in this study. We planned to conduct research with a large number of volunteers. However, there were excluded participants as follows: (1) bad quality of the images of MRA, and (2) volunteers whose blood samples had been collected one or more days after normal saline infusion. The other limitation was that hemodynamic changes might differ depending on the volume and types of the normal saline. However, the results of hemodynamic characteristics showed a statistically significant difference between the before and after groups. Thus, further study is needed with a large number of volunteers.

## 5. Conclusions

In the internal carotid artery, after infusion of intravenous normal saline, a recirculation zone with low intensity appeared at the regions with increased shear rate and reduced wall shear stress. At the carotid bifurcation, the after group showed an increased velocity and shear rate compared to the before group. Through the participant- and site-specific hemodynamic analysis between the two groups, injection of normal saline affected not only the reduction of blood viscosity, but also the overall changes of blood flow in the carotid artery. The accurate hemodynamic parameters could be obtained by applying the measured non-Newtonian blood viscosity to CFD study. Therefore, we found the possibility of improving blood flow because wall shear stress affecting on blood vessel was reduced by lowering the viscosity with the injection of normal saline.

## Figures and Tables

**Table 1:** Difference in the shear rate between before and after normal saline injection.

Arteries	Location	Shear Rates	Before	After	*p*
Whole Carotid Artery	Left	Minimal shear rate [s^−1^]	26.65 ± 4.73	28.64 ± 5.50	0.161
		Time-averaged shear rate [s^−1^]	186.96 ± 49.43	190.30 ± 46.43	0.263
		Maximal shear rate [s^−1^]	919.31 ± 236.19	935.52 ± 217.66	0.263
	Right	Minimal shear rate [s^−1^]	28.48 ± 4.10	32.05 ± 4.45	0.036 *
		Time-averaged shear rate [s^−1^]	212.89 ± 57.16	219.49 ± 54.55	0.050
		Maximal shear rate [s^−1^]	1043.55 ± 298.34	1074.73 ± 283.64	0.050
Internal Carotid Artery	Left	Minimal shear rate [s^−1^]	8.29 ± 2.87	8.69 ± 2.25	0.327
		Time-averaged shear rate [s^−1^]	91.87 ± 21.29	96.54 ± 20.01	0.012 *
		Maximal shear rate [s^−1^]	433.66 ± 103.99	459.23 ± 106.05	0.012 *
	Right	Minimal shear rate [s^−1^]	9.70 ± 5.48	10.17 ± 4.76	0.889
		Time-averaged shear rate [s^−1^]	89.66 ± 32.36	101.51 ± 41.31	0.036 *
		Maximal shear rate [s^−1^]	461.30 ± 225.85	483.00 ± 233.93	0.050

* indicates *p* < 0.05.

**Table 2:** Difference in the wall shear stress (WSS) between before and after normal saline injection.

Arteries	Location	Wall Shear Stresses	Before	After	*p*
Whole Carotid Artery	Left	Minimal WSS [Pa]	0.19 ± 0.02	0.18 ± 0.02	0.024 *
		Time-averaged WSS [Pa]	0.92 ± 0.18	0.84 ± 0.19	0.025 *
		Maximal WSS [Pa]	4.05 ± 0.81	3.74 ± 0.88	0.043 *
	Right	Minimal WSS [Pa]	0.20 ± 0.02	0.19 ± 0.02	0.020 *
		Time-averaged WSS [Pa]	1.04 ± 0.23	0.96 ± 0.22	0.043 *
		Maximal WSS [Pa]	4.58 ± 1.14	4.24 ± 1.07	0.036 *
Internal Carotid Artery	Left	Minimal WSS [Pa]	0.09 ± 0.01	0.08 ± 0.02	0.034 *
		Time-averaged WSS [Pa]	0.50 ± 0.07	0.46 ± 0.07	0.058
		Maximal WSS [Pa]	2.02 ± 0.33	1.91 ± 0.35	0.237
	Right	Minimal WSS [Pa]	0.09 ± 0.03	0.08 ± 0.03	0.034 *
		Time-averaged WSS [Pa]	0.52 ± 0.18	0.48 ± 0.17	0.025 *
		Maximal WSS [Pa]	2.14 ± 0.92	2.00 ± 0.88	0.025 *

* indicates *p* < 0.05.

## References

[1] ReferencesHa Y.K., Hong H., Yeom E., Song J.M. (2020). Numerical study of the pulsatile flow depending on non-Newtonian viscosity in a stenosed microchannel. J. Vis..

[2] Carty G., Chatpun S., Espino D.M. (2016). Modeling blood flow through intracranial aneurysms: A comparison of Newtonian and non-Newtonian viscosity. J. Med. Biol. Eng..

[3] Myburgh J.A., Mythen M.G. (2013). Resuscitation fluids. N. Engl. J. Med..

[4] Blumberg N., Cholette J.M., Pietropaoli A.P., Phipps R., Spinelli S.L., Eaton M.P., Noronha S.A., Seghatchian J., Heal J.M., Refaai M.A. (2018). 0.9% NaCl (Normal Saline)-Perhaps not so normal after all?. Transfus. Apher. Sci..

[5] Williams E.L., Hildebrand K.L., McCormick S.A., Bedel M.J. (1999). The effect of intravenous lactated Ringer’s solution versus 0.9% sodium chloride solution on serum osmolality in human volunteers. Anesth. Analg..

[6] Chowdhury A.H., Cox E.F., Francis S.T., Lobo D.N. (2012). A randomized, controlled, double-blind crossover study on the effects of 2-L infusions of 0.9% saline and plasma-lyte(R) 148 on renal blood flow velocity and renal cortical tissue perfusion in healthy volunteers. Ann. Surg..

[7] De Bruyne B., Adjedj J., Xaplanteris P., Ferrara A., Mo Y., Penicka M., Flore V., Pellicano M., Toth G., Barbato E. (2017). Saline-Induced Coronary Hyperemia: Mechanisms and Effects on Left Ventricular Function. Circ. Cardiovasc. Interv..

[8] Cecchi E., Giglioli C., Valente S., Lazzeri C., Gensini G.F., Abbate R., Mannini L. (2011). Role of hemodynamic shear stress in cardiovascular disease. Atherosclerosis.

[9] Li C.H., Gao B.L., Wang J.W., Liu J.F., Li H., Yang S.T. (2019). Hemodynamic Factors Affecting Carotid Sinus Atherosclerotic Stenosis. World Neurosurg..

[10] Li X., Sun B., Zhao H., Ge X., Liang F., Li X., Xu J., Liu X. (2018). Retrospective Study of Hemodynamic Changes Before and After Carotid Stenosis Formation by Vessel Surface Repairing. Sci. Rep..

[11] Birchall D., Zaman A., Hacker J., Davies G., Mendelow D. (2006). Analysis of haemodynamic disturbance in the atherosclerotic carotid artery using computational fluid dynamics. Eur. Radiol..

[12] Lee S.W., Antiga L., Spence J.D., Steinman D.A. (2008). Geometry of the carotid bifurcation predicts its exposure to disturbed flow. Stroke.

[13] Box F.M., van der Geest R.J., Rutten M.C., Reiber J.H. (2005). The influence of flow, vessel diameter, and non-newtonian blood viscosity on the wall shear stress in a carotid bifurcation model for unsteady flow. Invest. Radiol..

[14] Benzakoun J., Roca P., Calvet D., Naggara O., Lion S., Gobin-Metteil M.-P., Charron S., Cavero V., Meder J.-F., Edjlali M. (2019). Optimal 4DFlow MR sequence parameters for the assessment of internal carotid artery stenosis: A simulation study. Neuroradiology.

[15] Töger J., Zahr M.J., Aristokleous N., Markenroth Bloch K., Carlsson M., Persson P.O. (2020). Blood flow imaging by optimal matching of computational fluid dynamics to 4D-flow data. Magn. Reson. Med..

[16] Berman S.E., Rivera-Rivera L.A., Clark L.R., Racine A.M., Keevil J.G., Bratzke L.C., Carlsson C.M., Bendlin B.B., Rowley H.A., Blennow K. (2015). Intracranial arterial four-dimensional flow is associated with metrics of brain health and Alzheimer’s disease. Alzheimer’s Dement. Diagn. Assess. Dis. Monit..

[17] Oshida S., Mori F., Sasaki M., Sato Y., Kobayshi M., Yoshida K., Fujiwara S., Ogasawara K. (2018). Wall shear stress and T1 contrast ratio are associated with embolic signals during carotid exposure in endarterectomy. Stroke.

[18] Watanabe T., Isoda H., Takehara Y., Terada M., Naito T., Kosugi T., Onishi Y., Tanoi C., Izumi T. (2018). Hemodynamic vascular biomarkers for initiation of paraclinoid internal carotid artery aneurysms using patient-specific computational fluid dynamic simulation based on magnetic resonance imaging. Neuroradiology.

[19] Sun L., Wang J., Li M., Li M., Zhu Y. (2020). The contribution of wall shear stress insult to the growth of small unruptured cerebral aneurysms in longitudinal 3D-TOF-MRA. J. Neurol. Sci..

[20] Khan M.O., Arana V.T., Rubbert C., Cornelius J.F., Fischer I., Bostelmann R., Mijderwijk H.-J., Turowski B., Steiger H.-J., May R. (2020). Association between aneurysm hemodynamics and wall enhancement on 3D vessel wall MRI. J. Neurosurg..

[21] Zhao S.Z., Xu X.Y., Hughes A.D., Thom S.A., Stanton A.V., Ariff B., Long Q. (2000). Blood flow and vessel mechanics in a physiologically realistic model of a human carotid arterial bifurcation. J. Biomech..

[22] Gijsen F.J., Allanic E., van de Vosse F.N., Janssen J.D. (1999). The influence of the non-Newtonian properties of blood on the flow in large arteries: Unsteady flow in a 90 degrees curved tube. J. Biomech..

[23] Perktold K., Resch M., Florian H. (1991). Pulsatile non-Newtonian flow characteristics in a three-dimensional human carotid bifurcation model. J. Biomech. Eng..

[24] Chen Z., Qin H., Liu J., Wu B., Cheng Z., Jiang Y., Liu L., Jing L., Leng X., Jing J. (2020). Characteristics of Wall Shear Stress and Pressure of Intracranial Atherosclerosis Analyzed by a Computational Fluid Dynamics Model: A Pilot Study. Front. Neurol..

[25] Dai Y., Lv P., Javadzadegan A., Tang X., Qian Y., Lin J. (2018). Hemodynamic analysis of carotid artery after endarterectomy: A preliminary and quantitative imaging study based on computational fluid dynamics and magnetic resonance angiography. Quant. Imaging Med. Surg..

[26] Domanin M., Gallo D., Vergara C., Biondetti P., Forzenigo L.V., Morbiducci U. (2019). Prediction of long term restenosis risk after surgery in the carotid bifurcation by hemodynamic and geometric analysis. Ann. Biomed. Eng..

[27] Du J., Wu G., Wu B., Liu C., Mai Z., Liu Y., Wang Y., Zhang P., Wu G., Liu J. (2020). The Hemodynamic Effect of Enhanced External Counterpulsation Treatment on Atherosclerotic Plaque in the Carotid Artery: A Framework of Patient-Specific Computational Fluid Dynamics Analysis. Cardiol. Res. Pract..

[28] Cibis M., Potters W.V., Selwaness M., Gijsen F.J., Franco O.H., Lorza A.M.A., de Bruijne M., Hofman A., van der Lugt A., Nederveen A.J. (2016). Relation between wall shear stress and carotid artery wall thickening MRI versus CFD. J. Biomech..

[29] Gharahi H., Zambrano B.A., Zhu D.C., DeMarco J.K., Baek S. (2016). Computational fluid dynamic simulation of human carotid artery bifurcation based on anatomy and volumetric blood flow rate measured with magnetic resonance imaging. Int. J. Adv. Eng. Sci. Appl. Math..

[30] Lee S.H., Kang S., Hur N., Jeong S.K. (2012). A fluid-structure interaction analysis on hemodynamics in carotid artery based on patient-specific clinical data. J. Mech. Sci. Technol..

[31] Qin Y., Wu J.H., Hu Q.M., Ghista D.N., Wong K.K.L. (2017). Computational evaluation of smoothed particle hydrodynamics for implementing blood flow modelling through CT reconstructed arteries. J. X-Ray Sci. Technol..

[32] Shojima M., Oshima M., Takagi K., Torii R., Hayakawa M., Katada K., Morita A., Kirino T. (2004). Magnitude and role of wall shear stress on cerebral aneurysm: Computational fluid dynamic study of 20 middle cerebral artery aneurysms. Stroke.

[33] Kojima M., Irie K., Fukuda T., Arai F., Hirose Y., Negoro M. (2012). The study of flow diversion effects on aneurysm using multiple enterprise stents and two flow diverters. Asian J. Neurosurg..

[34] Jung J.M., Lee D.H., Kim K.T., Choi M.S., Cho Y.G., Lee H.S., Choi S.I., Lee S.R., Kim D.S. (2014). Reference intervals for whole blood viscosity using the analytical performance-evaluated scanning capillary tube viscometer. Clin. Biochem..

[35] Lee U.Y., Chung G.H., Jung J., Kwak H.S. (2020). Size-Dependent Distribution of Patient-Specific Hemodynamic Factors in Unruptured Cerebral Aneurysms Using Computational Fluid Dynamics. Diagnostics.

[36] Holdsworth D.W., Norley C.J.D., Frayne R., Steinman D.A., Rutt B.K. (1999). Characterization of common carotid artery blood-flow waveforms in normal human subjects. Physiol. Meas..

[37] Zakrzewski A.M., Anthony B.W. (2018). Noninvasive Blood Pressure Estimation Using Ultrasound and Simple Finite Element Models. IEEE Trans. Biomed. Eng..

[38] Papaioannou T.G., Stefanadis C. (2005). Vascular wall shear stress: Basic principles and methods. Hell. J. Cardiol..

[39] Awad S., Allison S.P., Lobo D.N. (2008). The history of 0.9% saline. Clin. Nutr..

[40] Kampmeier T., Rehberg S., Ertmer C. (2014). Evolution of fluid therapy. Best Pract. Res. Clin. Anaesthesiol..

[41] Saho T., Onishi H. (2017). Quantitative analysis of effects of hemodynamic stress on temporal variations of cardiac phases in models of human carotid bulbs. Radiol. Phys. Technol..

[42] Long Q., Xu X.Y., Ariff B., Thom S.A., Hughes A.D., Stanton A.V. (2000). Reconstruction of blood flow patterns in a human carotid bifurcation: A combined CFD and MRI study. J. Magn. Reson. Imaging Off. J. Int. Soc. Magn. Reson. Med..

[43] Webb A.R., Nash G.B., Dormandy J.A., Bennett E.D. (1990). A Comparison of the Effects of Artificial Plasma Substitutes, Albumin and Saline Solutions on Invitro Apparent Blood-Viscosity. Clin. Hemorheol..

